# New Nanotechnologies for the Treatment and Repair of Skin Burns Infections

**DOI:** 10.3390/ijms21020393

**Published:** 2020-01-08

**Authors:** Eliana B. Souto, André F. Ribeiro, Maria I. Ferreira, Maria C. Teixeira, Andrea A. M. Shimojo, José L. Soriano, Beatriz C. Naveros, Alessandra Durazzo, Massimo Lucarini, Selma B. Souto, Antonello Santini

**Affiliations:** 1 Department of Pharmaceutical Technology, Faculty of Pharmacy, University of Coimbra (FFUC), Polo das Ciências da Saúde, Azinhaga de Santa Comba, 3000-548 Coimbra, Portugal; andre_f_ribeiro@hotmail.com (A.F.R.); ines.ferreira6556@gmail.com (M.I.F.); mceuteixeira1@gmail.com (M.C.T.); lshimojo51@gmail.com (A.A.M.S.); 2 CEB—Centre of Biological Engineering, University of Minho, Campus de Gualtar 4710-057 Braga, Portugal; 3 Department of Engineering of Materials and Bioprocesses, School of Chemical Engineering, University of Campinas, Campinas 13083-852, Brazil; 4 Department of Pharmacy and Pharmaceutical Technology, Faculty of Pharmacy, University of Granada, 18071 Granada, Spain; jlsoriano@correo.ugr.es (J.L.S.); beatrizclares@ugr.es (B.C.N.); 5 CREA—Research Centre for Food and Nutrition, Via Ardeatina 546, 00178 Rome, Italy; alessandra.durazzo@crea.gov.it (A.D.); massimo.lucarini@crea.gov.it (M.L.); 6 Department of Endocrinology of Hospital de São João, Alameda Prof. Hernâni Monteiro, 4200-319 Porto, Portugal; sbsouto.md@gmail.com; 7 Department of Pharmacy, University of Napoli Federico II, Via D. Montesano 49, 80131 Napoli, Italy

**Keywords:** burn wound infection, antibiotics, wound healing, nanoparticles

## Abstract

Burn wounds are highly debilitating injuries, with significant morbidity and mortality rates worldwide. In association with the damage of the skin integrity, the risk of infection is increased, posing an obstacle to healing and potentially leading to sepsis. Another limitation against healing is associated with antibiotic resistance mainly due to the use of systemic antibiotics for the treatment of localized infections. Nanotechnology has been successful in finding strategies to incorporate antibiotics in nanoparticles for the treatment of local wounds, thereby avoiding the systemic exposure to the drug. This review focuses on the most recent advances on the use of nanoparticles in wound dressing formulations and in tissue engineering for the treatment of burn wound infections.

## 1. Introduction

Burn injuries are one of the most frequent causes of trauma, leading to morbidity and mortality mostly in low and middle-income countries. Burns can be caused by the (i) contact to high temperature materials (e.g., contact with fire or hot liquids, solids or gases), or by the (ii) exposure to cold environments, chemical agents (e.g., strong acids or bases), electricity or radiation exposure (e.g., ultra-violet light, X-ray, microwaves and others) [1]. Scald injuries are the most common type of burns in children, adults, and elderly patients [2].

Burns affect the integrity of the skin as its protective function is significantly compromised, allowing microbial invasion to occur, increasing the risk of localized colonization and systemic entry of microbes from both endogenous and exogenous origins [3]. According to the World Health Organization (WHO), burn injuries comprise over an estimated 180,000 annual deaths, representing a global public health problem [4]. In the Western world, the mortality rate due to burns has been declining, for example in England and Wales the rate is approximately 1.4% of all cases [2]. Per year, in Europe, the hospitalization rate is 2–29 hospitalizations per 100,000 inhabitants, and these injuries have a high impact, at the economic level, because they lead to long hospitalization periods and entail many costs. In Portugal, the latest surveys showed that the hospitalization rate was 18.9 hospitalizations/100,000 inhabitants/year between 2000–2013. This study is one of the largest southern European nationwide epidemiological study of burn patients and allowed to conclude that burn admissions are decreasing significantly [5].

Some groups, such as patients with diabetic neuropathy, patients with mental disorders, and children, are more vulnerable to these injuries [2]. In the case of children, there is a special care to be taken, because burns are the 11th cause of death in children between 1–9 years old and are the 5th cause of non-fatal injuries. Burns have a high impact on patients, both at physical level (due to disfigurement or disability), and at emotional level [6].

The survival rate has been increasing mainly because of improvements in various therapies such as pulmonary care, nutritional support, burn wound care and others. However, even though burn wounds are initially sterile in comparison to other acute skin injuries, in patients with more than 40% of the total body surface area (TBSA) burned, 75% of deaths are due to sepsis [7]. Thus, it is important to ascertain what bacteria are present in burn wounds and their resistance [8].

Burn wounds differ from any of the other traumatic wounds, being the immunosuppression the major cause of sepsis from invasive burn wound. Due to the increased capillary permeability, a high loss of plasma is observed in extensive burn wounds, which may lead to shock. Chemotactic, phagocytic and intracellular killing events are observed, together with the depression of cytokinin release and cell-mediated immune response, thereby reducing immunoglobulin serum levels, and compromising B-cell differentiation into antibody secreting cells. The highest incidence of septicaemia in burns occurs within the first 10 days, when serum immunoglobulin titres are markedly deranged [7]. The microorganisms that may cause invasive burn wound infection are summarized in Table 1.

In order to prevent the incidence of infection and sepsis, new therapeutic approaches are required, and nanomedicine is an area that can be extensively explored [9]. As the application of nanotechnology in medical care, nanomedicine is a promising area as it combines an improvement in health care with a reduction of the costs of therapy. In Europe, only a few nanomedicines have yet been approved when compared to the USA [10]. The approval of nanomedicines has encountered several obstacles attributed to the difficulty of fully describing the mechanism of action and interaction of nanomedicines with the biological systems, and also of scaling-up of manufacturing and quality control methods that are important for batch to batch uniformity [11,12,13]. The European Medicines Agency (EMA) may be open to new authorizations because clinical trials with nanomedicines have being increasing in Europe in the last decades. Studies on follow up, use, compliance, and assessment in this area are nevertheless still needed [14,15,16,17]. Regenerative medicine is one of the branches of nanomedicine that is most promising for the approval of new medicines and therapies [10].

Nanomedicine has the potential to improve treatments for non-curable diseases or diseases with poor prognosis, such as cancer, neurodegenerative diseases or infections. Nanosystems have been firstly proposed as carriers for drug delivery and targeting, but today nanomaterials themselves can also exhibit therapeutic properties [18]. As the most newly developed pharmaceuticals and nanonutraceuticals are poorly soluble drugs [19,20,21,22,23,24,25,26,27,28,29,30,31,32,33], the emerging field of nanotechnology presents as a very promising therapeutic alternative for topical wound healing treatment with its underlying target delivery mechanisms [9,34]. This review discusses the latest advances in nanomedicines and their relevance and advantages for the treatment of burn wound infections.

## 2. Pathophysiology of Burn Wound Infections

**Figure 1:**
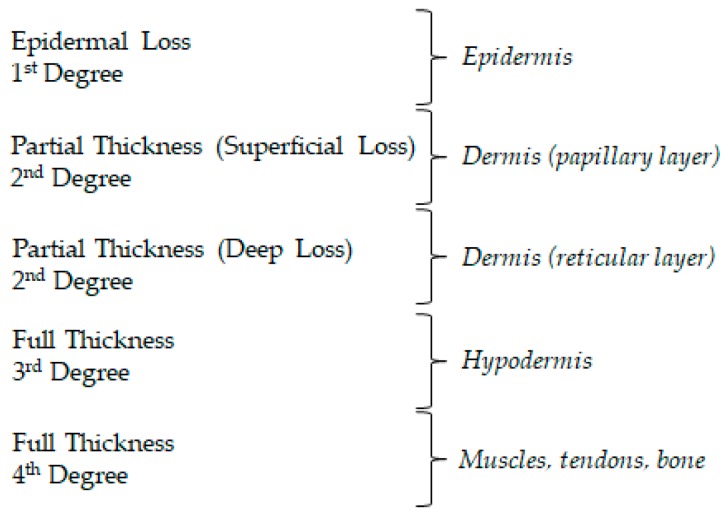
Different degrees of burn wounds and affected skin layers.

An injured skin derived from a burn wound can be classified according to the depth of the thermal damage (classified into four degrees) and the estimation of percentage of total body surface area (TBSA) that has been affected (determined by the rule of nines) [1]. The susceptibility to infection is highly influenced by the severity of the damage and may cause, in some more advanced cases, severe lesions to the underlying tissues of the skin. The depth of thermal skin damage is represented in Figure 1.

The skin is composed by three primary layers, videlicet epidermis, dermis, and hypodermis. The epidermis is the most external layer that serves as a skin protective barrier against external aggressions and maintains skin hydration and regeneration. Corneocytes, keratinocytes, melanocytes, Langerhans cells, Merkel cells, CD8+ T cells and stem cells are present in epidermis, among which Langerhans cells stand out by its function of antigen presenting cell within the adaptive immune system. The dermis is an irrigated fibrous matrix of collagen, elastin, nerve structures, cutaneous appendages and connective tissue [35], providing structural support, glandular function and irrigation [1]. Adipocytes, macrophages, fibroblasts, vascular, and nerve systems form the hypodermis and support the above-mentioned layers of tissue.

When skin integrity is compromised by wound injury, a restorative response for cutaneous repair takes place, involving four stages of repair: haemostasis, inflammation, proliferation, and remodelling. In the first stage, vasoconstriction, platelet aggregation and immunity, and complement system activation occur, ceasing the bleeding and activating the coagulation cascade. The clot formation aims to prevent the microorganism invasion. Simultaneously, the inflammation is installed, as the fibrin clot secrets cytokines and growth factors that induce the migration of neutrophils followed by macrophages (differentiated lymphocytes and monocytes) to the wound resulting in the production of reactive oxygen species (ROS) and proteinases, thereby protecting the organism against exogenous microorganisms [35]. The secretion of cytokines will promote a third stage, which upholds cells proliferation, angiogenesis, tissue granulation and ultimately promotes the formation of a temporary extracellular matrix. This mass is then replaced by a mature scar at the remodelling stage of the skin.

This regenerative process is potentially hampered by infection, which can be of fungal or bacterial origin, with the most prevalent microorganisms being *Staphylococcus aureus* (gram-positive bacteria), *Pseudomonas aeruginosa* [36,37] (gram-negative bacteria) and *Candida* spp. (fungi) [3]. Infection constitutes a major concern, as the production of a biofilm in the extracellular matrix protects the microorganisms against conventional antibiotics and is highly associated with hypertrophic scar [37]. After a burn injury, with the thermal destruction of the skin and the haemostasis phase, the burn wound surface takes the form of an avascular necrotic tissue with high protein content, which results in impaired immune cells migration and provides a favourable environment to microbial invasion and colonization. Gram-positive bacteria of endogenous origin that survive thermal stress, namely Staphylococci, are the first to colonize the burn wound within the first 48 h, followed by other pathogens [38]. The bacteria then initiate several mechanisms of virulent factors production, including host cell adhesion, immune system evasion, nutrient acquisition, which can lead to tissue destruction and systemic invasion if not rapidly intervened. The adhesion and proliferation will later promote the aggregate of microorganisms that secrete an extracellular matrix (slime) rich in proteins and polysaccharides, protecting the complex community from harsh environments in a biofilm.

Antibiotherapy, both topical and systemic, plays an important role in the prevention and treatment of wound infections. However, systemic antibiotics are highly associated with mechanisms of resistance, which compromise the treatment process. For local wound treatment, the deeper extension of the burn and increasing eschar thickness, the less reliable is the administration of systemic antibiotics to the eschar [39]. Therefore, topical antibiotics emerge as an alternative treatment, as these contribute to a “high and sustained concentration of the antimicrobial at the site of the infection” [40]. Despite their potential benefits, the results obtained with topical antibiotherapy are still “suboptimal” [41], which reinforces the need of innovation in topical antibiotic delivery systems.

## 3. Innovative Nanoparticles for the Treatment and Repair of Burn Wounds

For several decades, antibiotherapy has been successfully used in promoting human health, associated however with the increase of bacterial resistance [42]. Local antimicrobial treatment may be a suitable approach to overcome the risk of bacterial resistance as it reduces drug systemic exposure [43]. A range of new drug delivery systems has been proposed to allow the local administration of antibiotics [43]. Currently available therapeutic options include biological-based approaches for burn wound healing (immune-based antimicrobial molecules, reactive oxygen species and nitric oxide generators, antimicrobial agents and therapeutic microorganisms), antimicrobial light and ultrasound-based wound therapy (phototherapy and shockwave and ultrasound based-therapy), stem cell based-burn therapy and nanoparticles for the treatment of burn infections and wound healing [1]. Other options include surgical approaches, skin grafting and skin substitutes, wound dressings, negative pressure wound therapy (NPWT), pharmacological approaches, and skin tissue engineering [1,34,44]. The interaction of nanoparticles with the skin, and therefore their life-time in the site of action, is governed not only by the degree of injury of the skin but also by the type and size of nanoparticles. Injured and inflamed skin shows loss of its barrier function and higher risk of nanoparticles absorption to the systemic circulation and can even be entered by particles larger than the nanometer scale [45]. The ideal nanocarrier for the topical administration of drugs to skin wounds should be biodegradable, non-toxic and non-immunogenic, and posess the capacity to release the drug in a controlled fashion. The therapeutic effects should only be limited to the site of action and no systemic absorption should be observed. More important than the half-life of the nanoparticles onto the site of action (bio-resistant versus biodegradable nanoparticles) is their residence time in the wound which also depends on the cleaning rituals. The residence time of the nanocarrier onto the skin should also been increased attributed to the mucoadhesive properties of the nanocarrier’s matrix and small size. The matrix composition (e.g., natural versus synthetic sources) is instrumental for its metabolic and biodegradation profiles to limit the risk of adverse side effects. The size and type of nanoparticles should be selected to minimize the systemic absorption, circumventing the pharmacological activity to the wound.

### 3.1. Nanosystems for Antimicrobials Delivery

#### 3.1.1. Nanoemulsions (NE)

NEs constute a heterogeneous system of two immiscible liquids stabilized by a suitable surfactant [32,46,47,48,49,50,51]. The droplet diameter is usually between 20 and 400 nm and the limit must be less than 500 nm [52]. NE are a transparent and isotropic dispersion that can be classified in three categories: water-in-oil (w/o) dispersions, oil-in-water (o/w) dispersions and bicontinuous/multiple emulsion [52]. NEs have many advantages as they are nontoxic and non-irritant [32,46,53], can be integrated into various types of pharmaceutical forms (foams, liquids, creams, and sprays) [54], have long-term physical stability which reduces phenomena like creaming, sedimentation, and coalescence [55], can be used for parenteral and non-parenteral routes as skin and mucosa [54], allow the solubilization of hydrophilic and hydrophobic active ingredients [52], and frequently the Brownian motion is enough to compensate the kinetic instability caused by viscosity or gravity [55]. Some disadvantages may however be pointed out because the stability of NEs is also dependent on the surface electrical charge, while the size of of NEs can increase by a phenomenon called Ostwald ripening [54].

Bonferoni et al. [56] loaded α-tocopherol (αTph) in NEs composed of chitosan oleate (CS-OA) for treatment of skin wounds. Both chitosan and oleic acid are known for their positive effects in wound healing. The authors reported that CS-OA and αTph NEs promote cell proliferation in keratinocytes and fibroblast cell cultures. The study suggested that CS-OA and αTph are appropriate for topical application in wound healing [57].

Song et al. evaluated the antibacterial and anti-biofilm activity of a chlorhexidine acetate NE (CNE) against skin burn wound methicillin-resistant *Staphylococcus aureus* (MRSA) infections. The results showed that in comparison to the aqueous solution of the same drug, CNE had a better and faster action against MRSA and was more efficient at anti-biofilm activity (inhibiting biofilm formation and clearing the biofilm). Furthermore, CNE intensely disrupted cell walls and membranes of MRSA [58].

#### 3.1.2. Polymeric Nanoparticles

Polymeric nanoparticles can be produced from natural, semi-synthetic or synthetic polymers to obtain particles within the range of 10–1000 nm. Depending on the type of polymer, drugs can be encapsulated (either dissolved or dispersed) within the nanoparticle matrix or attached onto its surface (chemically bound). The ability of polymeric nanoparticles to permeate skin layers depends on their chemical composition, the particle size, and the viscosity of the formulation [54]. They have several advantages such as easy manufacturing, physical and chemical stability [59], diversity of sizes and shapes [60], high drug loading capacity, and the possibility of controlled drug release and targeting [59].

Natural polymers exhibit biocompatibility and biodegradability but have some drawbacks such as batch variability and the risk of being immunogenic. Synthetic polymers, as they have known chemical composition, make it easier to predict their properties [61].

Regarding natural polymers, chitosan has been extensively studied in this field. Chitosan is a polysaccharide formed by a randomly distributed β-(1→4)-linked *D*-glucosamine and *N*-acetyl-*D*-glucosamine monomers. The chitosan types differ in molecular weight, degree and pattern of *N*-acetylation [62]. They are obtained by the process of alkaline deacetylation of chitin [63]. Chitin is found in the exoskeleton of crustaceans, insects and fungal cell walls [64]. The chitosan pKa is approximately 6.5, being soluble in acid medium and insoluble in alkaline and neutral media [63]. Chitosan has been investigated for use in several areas such as food, cosmetic, pharmaceutical and agrochemical industries [34,44,62,65,66,67]. Chitosan is non-toxic, biocompatible, biodegradable [63], has antimicrobial activity, and low immunogenicity while properties like mucoadhesion, controlled drug delivery, and in situ gelation improve permeation [68]. The antimicrobial activity of chitosan is attributed to several mechanisms, namely, (i) interference of the chitosan molecules (which have a positive charge) in the bacterial surface (which has a negative charge) by modifying the permeability of the cell membrane, (ii) inhibition of the mRNA and protein synthesis by hydrolysis products, (iii) restriction of cell growth, by forming chelates with nutrients and essential metals, and (iv) inhibition of oxygen (essential to the growth of aerobic bacteria) and nutrients by a polymer membrane which surrounds the surface of the cell [69].

Chitosan has been associated with the ability to improve wound healing. because it enhances the activity of fibroblasts, macrophages and polymorphonuclear leukocytes [70].

Nguyen et al. incorporated fibroblast growth factor 2 (FGF-2), a protein that participates in wound healing but highly unstable in vivo, in carboxylmethyl chitosan (CMCS) nanoparticles [71]. Nanoparticles composed of CMCS:CaCl_2_ (ratio of 1:0.8) with 95% of encapsulation efficiency for FGF-2 were produced using CaCl_2_ as crosslinking agent. Particles were incubated in pH 7.4 and 5.8 media for 48 h, during which 36.36% and 58.47% release of FGF-2, respectively, were recorded. The obtained results reveal that in vitro degradation of FGF-2 by trypsin could be avoided by the loading of the protein into the CMCS nanoparticles, and that the activity of FGF-2 was not compromised by the encapsulation and release process.

Ding et al. described the cross-linking of chitosan with genipin following the adding of partially oxidized *Bletilla striata* polysaccharide to prepare a new biomaterial for the treatment of wounds (CSGB) [72]. The developed material exhibited lower gelation time, increased water retention and increased proliferation of L929 cells when compared to chitosan cross-linked only with genipin. The CSGB has however practically lost the antibacterial action because the amino groups of chitosan were partially inhibited. To overcome this problem, the authors prepared a bilayer composite of chitosan-silver nanoparticles (CS-AgG) on CSGB. This study allowed to conclude that either CSGB or bilayer wound dressing accelerated the wound healing in mice. The bilayer also showed enhanced epidermization with a decrease in inflammatory cells.

El-Feky et al. loaded silver sulfadiazine (SSD) in chitosan nanoparticles as a dressing for burn wound treatment [73]. The particles showed controlled release of SSD, which inhibited the proliferation of Gram-negative and Gram-positive bacteria and *Candida albicans* in an infected wound.

Blažević et al. developed melatonin-loaded lecithin/chitosan nanoparticles and and tested their effect on the wound epithelialisation in vitro [74]. Four different types of chitosan were tested for their biocompatibility and in vitro release of melatonin. The potential to promote wound epithelialization was evaluated by an in vitro scratch assay using a human keratinocyte (HaCaT) monolayer. A 5 μg/mL chitosan concentration of nanoparticles was tested on model wounds. The combination of chitosan and melatonin promoted wound epithelialization, while the effect on the proliferation and migration of keratinocytes was independent of the type of chitosan used for the production of nanoparticles.

#### 3.1.3. Metal Nanoparticles

Metal nanoparticles have been used for different purposes such as medical imaging, diagnostics, drug delivery, antimicrobial coatings and environmental sensors [75,76]. Their use in the treatment of infections is of great relevance because of the recognised antimicrobial properties associated with a high surface/volume ratio [77]. Silver nanoparticles have been extensively used for this purpose [76]. Silver is a shiny, greyish metal that has been used for medicinal purposes for many years. Formulations with AgNPs are more effective than when silver is used in its macroscopic counter-parts. Silver nanoparticles can be produced by physical methods (e.g., evaporation-condensation and laser ablation), chemical methods (e.g., chemical reduction with a reducing agent) or biological methods (e.g., synthesis by microorganisms) [78]. The antimicrobial properties of silver are due to the ability to disturb the plasma membrane, which alters the permeability, resulting in lysis of the microorganism. AgNPs may also bind to the DNA, preventing its replication, or bind to the ribosomes preventing protein synthesis [75].

Mehrabani et al. prepared scaffolds from silk fibroin/chitin/silver nanoparticles by freeze-drying [79]. The antimicrobial activity of the obtained scaffolds was evaluated against *Escherichia coli*, *Staphylococcus aureus*, and *Candida albicans*, and these were shown to be biocompatible (cytocompatible) and biodegradable, with high antimicrobial activity, blood clotting, and water-uptake capability.

Tabaii and Emtiazi produced transparent bacterial cellulose membranes impregnated with antimicrobial AgNPs [80]. The AgNPs showed a diameter between 20–60 nm, while the AgNPs/BC membranes exhibited long-term stability with a high antimicrobial activity against *Escherichia coli* and *Staphylococcus aureus*.

#### 3.1.4. Nanogels

Nanogels consist of hydrogels composed of particles within the range of nanometers [81]. Hydrogels are three-dimensional cross-linked polymeric networks and have the capacity to uptake high amount of water or biological fluid [82]. Their loading with drugs is spontaneous and the production does not require mechanical energy or organic solvents [83]. Nanogels are biocompatible and prevent the enzymatic degradation of the drug [81], but those produced from particles with smaller size than the pore meshes in nanogels have the risk of leakage from nanogels network [81].

El-Feky et al. produced several silver sulfadiazine loaded nanogels based on chitosan and alginate [84]. The in vitro release studies in phosphate buffer (pH 7.4) showed a burst release of the drug followed by a prolonged release over a period of 3 h. In comparison to marketed products of the same drug, the developed nanogels had greater therapeutic efficacy in vivo in a female rat model of burn wound.

### 3.2. Wound Dressings

Wounds can be classified as acute or chronic, and can be divided into subclasses, such as pressure ulcers, venous leg ulcers, diabetic foot ulcers, traumatic wounds and surgical wounds [34,44]. Wound dressings allow to cover the wound, accelerating the healing and protecting it from external aggressions, such as damages, desiccation and microorganisms [85]. An ideal dressing should remove the excess of the exudate, it should moisturize and protect the skin, allow a clean, warm and moist environment, together with an adequate gaseous exchange, easy to use and be cost-effective. In addition, dressings should not release fibers or particles from their composition, or cause pain upon application or removal [34,44,86].

Wound dressings may present different physical forms, such as sponges, films, membranes, hydrogels or hydrocolloids [87], and can be divided into two categories: (i) traditional wound dressing, or (ii) modern wound dressing. Traditional wound dressings are used as primary dressings on clean and dry wounds with little exudation or as secondary dressing. Some examples are gauzes, bandages and plasters. Most of these products have to be changed regularly and adhere easily to the wound causing difficulties at the time of removal. For these reasons, modern dressings are more used because they maintain a humid environment and accelerate cicatrisation. There is a great variety of modern wound dressings, most of them based on synthetic polymers [88].

It is usual to classify wound dressings into four classes: biological dressings, conventional dressings, biosynthetic dressings, and antimicrobial dressings [85]. The purpose of using antimicrobial dressings is to prevent bacterial infections in burn wounds that are more susceptible to invasion by microorganisms. There are several antimicrobial agents that can be used in wound dressings [85]. To choose the antimicrobial agent, one must consider its specificity and efficacy, the potential to induce resistance and its safety in terms of cytotoxicity and allergenicity [89]. Antibiotics, nanoparticles and natural products (e.g., chitosan) are examples of antimicrobial agents [87]. It is common to find antimicrobial dressings containing silver (ionic silver-impregnated dressings and silver sulfadiazine), iodine (povidone iodine and cadexomer iodine) or natural products (such as honey) that are debriding agent and can mask odours [85,89]. The use of silver compounds in the treatment of burns has a fundamental role in decreasing the number of cases of sepsis and death [85]. The period of use of antimicrobial products should be as short as possible, preferably less than two weeks [89].

Biswas et al. produced porous chitosan/PVA (CS) scaffolds (CS-Se and CS Ag) by an in situ deposition method to be loaded either with silver (Ag) or selenium (Se) to be used in wound dressings [90]. The activity of these two types of scaffolds has been evaluated against gram-positive bacterium (*Staphylococcus aureus*), gram-negative bacterium (*Escherichia coli*), and multi-resistant (methicillin-resistant *S. aureus*, MRSA) bacteria. The Ag-loaded scaffolds showed a strong bactericidal activity against the studied species, but caused cytotoxicity in fibroblasts. The Se-loaded scaffolds were shown to be membranolytic against the studied strains and were non-toxic towards fibroblast

Kaygusuz et al. compared the antibacterial properties of cerium (III) crosslinked alginate films (Ce films) and cerium (III)-chitosan crosslinked alginate films (Ce-Chi films), with conventional calcium alginate films, against *Escherichia coli* (Gram-negative bacteria) and *Staphylococcus aureus* (Gram-positive bacteria) [91]. The results showed that Ce and Ce-Chi films had higher antibacterial activity than films with calcium. Ce and Ce-Chi films also showed improved physical properties and flexibility. The study suggested that Ce-Chi films can be used in antibacterial wound dressings.

### 3.3. Tissue Engineering

Tissue engineering and regenerative medicine (TERM) is an interdisciplinary field that combines engineering and biology with clinical practice [92]. In acute and chronic thermal injuries, skin tissue engineering aims to enhance wound healing, trigger skin regeneration and reduce the long term consequences of scarring with the development of innovative biomaterials, advanced technologies (such as electrospinning, recombinant proteins, 3D printing) and new methods to incorporate autologous cells [85]. Scaffolds, cells and growth factors can be combined in the construction of three-dimensional structures for skin regeneration for the treatment of burn wound. For further illustrations, please refer to Ho et al. [93].

It has been reported that the use of alpha-gal nanoparticles, composed of glycolipids with alpha-gal epitopes, phospholipids, and cholesterol, to wounds has induced accelerated healing by harnessing the natural anti-Gal antibody [94]. The healing time was reduced by 40%–60%, associated to the increased tropism of macrophages, angiogenesis, as well as epidermis and dermis growth. It has also been reported that such an approach could significantly reduce the risk of fibrosis and formation of scars. Biomaterials used in tissue engineering can be of natural or synthetic polymeric nature. Natural biomaterials include commercial products based on collagen, hyaluronic acid, elastin, chitin and fibrin [85,95], which are favourable to skin tissue regeneration due to their mechanical similarity, biocompatibility and biodegradability properties. Synthetic polymers include polylactic acid and polyglycolic acid, which can be used as cell growth support in scaffolds [95].

Steffens et al. developed an acellular matrix of poly-d,l-lactic acid (PDLLA) scaffolds produced by the electrospinning technique, linked to laminin-332 protein (isoform α3β3γ2) [95]. The electrospinning technique, by submitting a polymeric solution to an electric field, allowed the formation of a solid fibrous structure that would mimic the extracellular matrix arrangement and support cell growth. The inclusion of laminin, as a cell adhesion natural protein, was based on its importance in keratinocyte migration. The skin substitute was posteriorly completed with the inclusion of a cellular component constituted by mesenchymal stem cells and keratinocytes, directed to the dermal and epidermal skin regeneration, respectively.

Several other innovative therapies for burn wound treatment have also been proposed, among which is the use of recombinant proteins [96], such as elastin-like polypeptides [97]. Elastin is a biocompatible natural human skin component with structural functions [97]. Based on hypertrophic scarring related to the augmented expression of collagen and low elastin fiber production, tropoelastin delivery aims to the improvement of skin flexibility in burn wound healing [98].

Xie et al. studied the impact subcutaneous injections of recombinant human tropoelastin in skin flexibility in partial thickness thermal wounds [98]. As a soluble precursor of elastin, tropoelastin was used to enable elastin fiber production in burn and surgical scars. The treatment evidenced the production of new elastin fibers in site, although no improvement in skin flexibility was observed [98].

Thermal injuries may represent extensive skin damage. Full-thickness burns are a major concern, as the full integrity of the skin has been completely compromised, to the point in which it has no capacity to repair autologously [99]. Situations like full-thickness burns (3rd and 4th degrees, Figure 1) require a skin substitute therapy, such as three-dimensional biocompatible structures to provide skin regeneration in burn wound healing [99].

Law et al. characterized hyaluronic acid methylcellulose (HAMC) hydrogels for 3D bioprinting [100]. The suitability of HAMC for cell-encapsulated three-dimensional bioprinting applications, adequate for scaffold structures and cell delivery, with mesenchymal stem cell viability for one week (minimum) in bioprinted structures has been confirmed [100].

Combining tissue engineering, regenerative medicine and 3D printing (by placing cells and extracellular matrix in tissue native microenvironments) with big data analysis is offering new perspectives in the treatment of burn wound patients [101].

Gholipourmalekabadi et al. developed a 3D bi-layer scaffold of biological decellularized human amniotic membrane (AM) with viscoelastic electrospun nanofibrous silk fibroin (ESF) [102]. Adipose tissue-derived mesenchymal stem cells (AT-MSCs), cultured for seven days on the AM-ESF scaffold, demonstrated the expression of vascular endothelial growth factor (VEGF) and basic fibroblast growth factor (bFGF), which suggests potential suitability for AM/ESF scaffold with autologous AT-MSCs [102].

Kuna et al. produced a novel composite gel from human peripheral blood mononuclear cells (hPBMCs) and decellularized gal-gal-knockout porcine skin for skin wound healing [103]. To obtain a pig skin gel (PSG), the prepared decellularized skin extracellular matrix (ECM) powder was mixed with culture medium containing hyaluronic acid. Nude mice were used to evaluate the effect of the composite in regeneration of wounds, showing a significant wound closure within 15 days of treatment with PSG only or with PSG containing hPBMCs. The addition of hPBMCs to PSG improved the host blood vessels network and induced the development of human blood vessels.

A bio-ink prepared using skin decellularized ECM has been recently described [104]. The bio-ink was obtained by combining decellularized ECM (containing collagen, glycosaminoglycans and growth factors and free from immune-reactive cells) with human dermal fibroblasts. The obtained construct showed viability over 90%, demonstrating skin regenerative effect of the bio-ink.

The delivery of growth factors to the burn wound was also studied as a tissue engineering technology, presenting beneficial effects in tissue repair. Liu et al. developed bFGF-loaded alginate microspheres (Ms) incorporated into carboxymethyl chitosan (CMCS)–poly(vinyl alcohol) (PVA) as a composite hydrogel, enabling basic fibroblast growth factor delivery without bioactivity loss [99]. The studies comprised a 2-weeks treatment in vivo in rats, in which improved wound recovery rates, with re-epithelialization and regeneration of the dermis was seen [99]. However, when addressing burn wound treatment, it is important to take in consideration that compromised skin tissue is greatly associated to functional limitations and infection susceptibility, which may affect the success of tissue engineering technologies.

To address these limitations, several studies have been carried out towards the development of an ideal wound dressing that would combine both skin regeneration and infection control, generally focused on silver antimicrobial and biosensor activities [105]. Table 2 summarizes some examples.

Zulkifli et al. developed a hydroxyethyl cellulose-silver nanoparticle (HEC-AgNP) lyophilized scaffold [106]. In this study, hydroxyethyl cellulose serves as a polymer matrix and a reducing agent of silver ions to a zero-valent form and nanoparticle formation using freeze-dry methodology. When in contact with moisture or wound fluid, silver ions are released, inhibiting bacterial growth, without toxicity effects on human fibroblast cell growth [106].

Advanced studies of AgNP delivery systems allowed not only the delivery of nanoparticles, but also the development and optimization of control release of the nanoparticles from the scaffold to the burn wound. Liu et al. studied the incorporation of silver nanoparticles on the surface of a natural biomaterial of collagens type I, V, and X (eggshell membrane) using polydopamine mediated adhesion and reduction properties, which exhibited benefits for tissue regeneration, biocompatibility, and low toxicity [105,107].

Despite the aforementioned technological progress and silver broad antimicrobial activity, there are some limitations associated with silver products, such as cytotoxicity to host cells (fibroblasts and keratinocytes) and emergence of bacterial resistant strains (e.g., *Escherichia coli*) [108]. Topical antibiotic delivery may comprise an alternative approach for the treatment of burn wound infection.

Chen et al. developed electrospun gelatin fibres with gentamicin sulfate and hydrophobic ciprofloxacin release for deep infected burns, with evidenced effectiveness against *Pseudomonas aeruginosa* and *Staphylococcus epidermidis* infections [107]. The electrospinning technique allowed the formation of a fibrous structure that would mimic the extracellular matrix, composed of a cross-linked fibre of denatured collagen derivative (gelatin) and alginate dialdehyde. This product was loaded with the antibiotics, allowing the prompt gentamicin sulfate delivery and ciprofloxacin sustained release, for initial and maintenance infection control, respectively. This combined system, demonstrated successful antibiotic release and accelerated dermal regeneration in burn wound infection [107].

## 4. Conclusions

In the past few years, several efforts have been made to improve the way wound infections are treated. Technological developments in the field of nanomedicines have been instrumental in finding new alternatives to overcome antimicrobial resistance against antibiotics Several types of nanoparticles have been proposed, e.g., nanoemulsions, polymeric nanoparticles, metal nanoparticles, nanogels, with the aim to enhance the antimicrobial activity of compounds. The choice of nanoparticle is governed by the type of drug and by the extend of injured skin. Wound dressings made from, e.g., sponges, films, membranes, hydrogels or hydrocolloids, have also been proposed to offer protection against invasive microorganisms and accelerate the healing process, while regenerative medicine and tissue engineering make use of scaffolds, cells and growth factors for the construction of three-dimensional structures for skin regeneration. Future perspectives in the treatment of wound burns encompass the use of mesenchyme stem cells and progenitor cells, combined with nanomedicines, as these cells are known to be involved in skin regeneration. The delivery of cytokines, growth factors, and inflammatory mediators may further improve the healing process contributing for a synergistic effect in the treatment of wound burns. 

## Figures and Tables

**Table 1:** Microorganisms that cause invasive burn wound infections.

Group	Species
Gram-positive organisms	*Staphylococcus aureus*
	Methicillin-resistant *Staphylococcus aureus* (MRSA)
	Coagulase-negative staphylococci
	*Enterococcus* spp.
	Vancomycin-resistant enterococci
Gram-negative organisms	*Pseudomonas aeruginosa*
	*Escherichia coli*
	*Klebsiella* *pneumoniae*
	*Enterobacter* spp.
	*Proteus* spp.
	*Acinetobacter* spp.
	*Bacteroides* spp.
Fungi	*Candida* spp.
	*Aspergillus* spp.
Viruses	Herpes simplex
	Cytomegalovirus
	Varicella-zoster virus

**Table 2:** Summary of the recent studies on nanosystems, wound dressings and tissue engineering for burn wound infection.

**Recent Studies on Nanoparticles for Antimicrobials Delivery**			
**System**	**Antimicrobial Compounds**	**Study Description**	**References**
**Nanoemulsions (NEs)**			
Chitosan oleate and α-tocopherol NEs	α-tocopherol and chitosan	Encapsulation of α-tocopherol by chitosan oleate. The results indicated that both compounds promote cell proliferation on keratinocytes and fibroblast cell cultures.	[56]
Chlorhexidine acetate NEs (CNE)	Chlorhexidine acetate	Evaluation of the antibacterial and anti-biofilm activity of CNE against methicillin-resistant Staphylococcus aureus infections.	[58]
**Polymeric nanoparticles**			
Carboxylmethylchitosan nanoparticles (CMCS-NPs)	-	Incorporation of fibroblast growth factor 2 in CMCS:CaCl_2_ NPs. The results show that the nanoparticles were able to avoid the destruction of FGF-2 by trypsin.	[71]
Carbohydrate Polymers	*Bletillastriata* polysaccharide and genipin	Evaluation of the various physico-chemical and biological characteristics of partially oxidized *Bletillastriata* polysaccharide to chitosan cross-linked with genipin.	[72]
Silver sulfadiazine (SSD) loaded chitosan nanoparticles (CSNPs)	Silver sulfadiazine	Particle optimization and characterization of physical properties, antibacterial efficacy and fungicidal activity for the dressing with silver sulfadiazine (SSD) loaded chitosan nanoparticles (CSNPs). Results shown inhibition of the proliferation of Gram negative and Gram-positive bacteria and *Candida albicans.*	[73]
Melatonin-loaded lecithin/chitosan nanoparticles	-	Preparation of nanoparticles with four different types of chitosan. Nanoparticles characterization and biocompatibility and study of the in vitro release of melatonin.	[74]
**Metallic nanoparticles**			
Fibroin/chitin/silver nanoparticles	Silver	Preparation of silk fibroin/chitin/silver nanoparticles scaffolds by freeze-drying method, characterization and antimicrobial activity against *Escherichia coli*, *Staphylococcus aureus*, and *Candida albicans.*	[79]
Silver nanoparticles	Silver	Preparation of antimicrobial silver nanoparticles/bacterial cellulose (AgNPs/BC) membranes and their characterization, biocompatibility and antimicrobial activity. The results shown good antimicrobial activity against *Escherichia coli* and *Staphylococcus aureus.*	[80]
**Nanogels**			
Silver sulfadiazine nanogel	Silver sulfadiazine	Optimization and characterization of several silver sulfadiazine loaded nanogel formulations.	[84]
**Recent studies on wound dressings**			
**System**	**Antimicrobial Compounds**	**Study Description**	**References**
Silver-loaded scaffolds and Selenium-loaded scaffolds	Silver and selenium	Incorporation of silver and selenium separately into porous chitosan/PVA scaffolds by in situ deposition method. Characterization of scaffolds with Se or Ag nanostructures respectively, and antimicrobial activity against *Staphylococcus aureus, Escherichia coli*, and *Methicillin-Resistant S. Aureus*.	[90]
Cerium(III)crosslinked alginate films and cerium(III)-chitosan crosslinked alginate films	Cerium	Preparation of crosslinked alginate films and cerium (III)-chitosan crosslinked alginate films, characterization and comparation to physical and antibacterial properties of conventional calcium alginate films. Test of antimicrobial activity in *Escherichia coli* and *Staphylococcus aureus*.	[91]
**Tissue engineering**			
**System**	**Antimicrobial Compounds**	**Study Description**	**References**
Acellular matrix of poly-d,l-lactic acid scaffolds	-	Development of an acellular matrix of poly-d,l-lactic acid (PDLLA) scaffolds produced by the electrospinning technique, linked to laminin-332 protein (isoform α3β3γ2), completed with the inclusion of a cellular component constituted by mesenchymal stem cells and keratinocytes.	[95]
Subcutaneous injections of recombinant human tropoelastin	-	The impact of subcutaneous injections of recombinant human tropoelastin in skin flexibility, in partial thickness thermal wounds.	[98]
Methylcellulose Hydrogels for cell-encapsulated 3D bioprinting	-	Characterization of hyaluronic acid methylcellulose (HAMC) hydrogels for 3D bioprinting, adequate for scaffold structures and cell delivery.	[100]
3D bi-layer scaffold of biological decellularized human amniotic membrane (AM) with viscoelastic electrospun nanofibrous silk fibroin (ESF) and incorporated adipose tissue-derived mesenchymal stem cells.	-	Development of a 3D bi-layer scaffold of biological decellularized human amniotic membrane (AM) with viscoelastic electrospun nanofibrous silk fibroin (ESF). Adipose tissue-derived mesenchymal stem cells (AT-MSCs) cultured for seven days on the AM-ESF scaffold, demonstrated the expression of vascular endothelial growth factor (VEGF) and basic fibroblast growth factor (bFGF).	[102]
bFGF-loaded alginate microspheres (Ms) incorporated into a carboxymethyl chitosan-poly(vinyl alcohol) hydrogel.	-	Development of bFGF-loaded alginate microspheres (Ms) incorporated into carboxymethyl chitosan (CMCS)–poly(vinyl alcohol) (PVA) as a composite hydrogel.	[99]
Hydroxyethyl cellulose-silver nanoparticle (HEC-AgNP) lyophilized scaffold	Silver nanoparticles	Development of a hydroxyethyl cellulose-silver nanoparticle (HEC-AgNP) lyophilized scaffold, using freeze-dry methodology.	[106]
Polydopamine and collagens type I, V, X eggshell membrane loaded with silver nanoparticles	Silver nanoparticles	Study of the incorporation of silver nanoparticles on the surface of a natural biomaterial of collagens type I, V and X (eggshell membrane) using polydopamine mediated adhesion and reduction properties, which exhibited benefits for tissue regeneration, biocompatibility and low toxicity.	[107]
Crosslinked electrospun gelatin fibers loaded with gentamicin sulfate and ciprofloxacin	Gentamicin sulfate and ciprofloxacin	Development of crosslinked electrospun gelatin fibers loaded with gentamicin sulfate and hydrophobic ciprofloxacin release for deep infected burns, against *Pseudomonas aeruginosa* and *Staphylococcus epidermidis* infections.	[107]
